# AI-derived prognostic model identifies high-risk gene signatures in pediatric gliomas

**DOI:** 10.3389/fimmu.2026.1704720

**Published:** 2026-03-09

**Authors:** Ganglong Li, Fuyu Pei, Weizhen Wang

**Affiliations:** 1 Department of Pediatric Surgery, Guangdong Women and Children Hospital, Guangzhou, China; 2 Department of Pediatrics, Nanfang Hospital, Southern Medical University, Guangzhou, China; 3 Department of Ultrasound, Nanfang Hospital, Southern Medical University, Guangzhou, China

**Keywords:** artificial intelligence, machine learning, pediatric glioma, prognostic model, single-cell RNA sequencing

## Abstract

**Background:**

Pediatric gliomas, comprising both low-grade (LGGs) and high-grade gliomas (HGGs), exhibit significant molecular and clinical heterogeneity. While LGGs generally have a favorable prognosis, HGGs are associated with poor long-term survival despite aggressive treatment. Advances in molecular profiling have enabled targeted therapies, but treatment resistance and tumor heterogeneity remain major challenges. The integration of artificial intelligence (AI) and transcriptomic data holds promise for refining prognostic models and guiding personalized treatment strategies, yet its application in pediatric gliomas remains underexplored.

**Method:**

We applied the Artificial Intelligence-Derived Prognostic Index (AIDPI) model to analyze transcriptomic data from pediatric glioma patients. Differentially expressed genes (DEGs) were identified and incorporated into a machine learning-based prognostic model. Single-cell RNA-seq data were also integrated to assess cellular heterogeneity within the tumor microenvironment. Kaplan-Meier survival analysis, Cox regression, and receiver operating characteristic (ROC) curve analysis were performed to evaluate the model’s predictive power. Functional enrichment analysis was conducted to explore potential therapeutic targets.

**Results:**

The AIDPI model identified nine key genes (GRIA1, ZNF165, TM9SF2, PRKAR2A, PSMD6, H1F0, CDC25B, HIST1H2AE, and NCAPD2) that were consistently associated with prognosis across multiple pediatric glioma datasets. These genes were used to construct a machine learning-based prognostic model, which demonstrated superior predictive performance with a C-index > 0.85. High AIDPI scores correlated with poorer survival outcomes, as confirmed by Kaplan-Meier survival analysis and time-dependent ROC curves. The AIDPI model outperformed 30 other glioma prognostic models, highlighting its potential for precision prognosis. Functional analysis of the AIDPI-related genes revealed involvement in immune suppression and cell adhesion pathways. Single-cell analysis identified TM9SF2 and H1F0 as key prognostic genes, with high H1F0 expression being associated with poor prognosis in pediatric gliomas.

**Conclusions:**

Our findings highlight the potential of AI-driven transcriptomic analysis in improving pediatric glioma prognosis. The identified gene signatures may serve as biomarkers for risk stratification and personalized treatment strategies, advancing precision oncology in pediatric neuro-oncology.

## 1 Introduction

Gliomas represent nearly half of all pediatric central nervous system tumors and are broadly classified into low-grade gliomas (LGGs) and high-grade gliomas (HGGs) based on their histological and molecular characteristics (1). LGGs, which account for the majority of pediatric gliomas, are generally associated with a favorable prognosis, with an overall survival rate exceeding 90% (2). In contrast, HGGs constitute approximately one-third of pediatric gliomas and exhibit an extremely poor prognosis, with less than 10% long-term survival, despite intensive multimodal treatment strategies (3). Although advancements in diagnosis and therapeutic interventions have improved the overall survival rates for pediatric glioma patients, long-term clinical outcomes remain suboptimal, with survivors often experiencing significant comorbidities, reduced quality of life, and an elevated risk of mortality (4). These challenges underscore the urgent need for improved prognostic models and personalized treatment strategies.

Recent genomic and transcriptomic analyses have provided valuable insights into the molecular underpinnings of pediatric gliomas, paving the way for targeted therapies. The identification of genetic aberrations, such as BRAF mutations and fusions in LGGs, has facilitated the development of BRAF and MEK inhibitors, which have demonstrated promising clinical efficacy (5, 6). Despite these advancements, several challenges persist, including treatment resistance, tumor heterogeneity, and the need for long-term monitoring of adverse effects. Given the genetic and molecular diversity of pediatric gliomas, a one-size-fits-all approach remains inadequate, necessitating personalized prognostic and therapeutic models to optimize patient outcomes.

Concurrently, artificial intelligence (AI) and machine learning (ML) techniques have shown great promise in refining prognostic models and clinical decision-making (7). AI-driven approaches, such as multiparametric radiomics and deep-learning-based histological classification, have been utilized to improve glioma grading and predict patient survival (8). Moreover, ML models leveraging diffusion kurtosis imaging and clinical parameters have demonstrated superior performance in prognostic assessments (9). While these methodologies have significantly enhanced diagnostic accuracy, their integration with transcriptomic-based predictive models remains an underexplored avenue in pediatric glioma research.

In this study, we aim to evaluate the prognostic significance of a novel gene signature derived from the Artificial Intelligence-Derived Prognostic Index (AIDPI) model in pediatric gliomas. By integrating large-scale transcriptomic datasets with machine learning approaches, we seek to refine prognostic stratification, identify key molecular drivers associated with survival, and provide insights for personalized therapeutic strategies. Our findings offer a computationally driven framework that could enhance precision medicine approaches for pediatric glioma patients.

## 2 Materials and methods

### 2.1 Acquisition and processing of transcriptomic data

RNA expression profiles and corresponding clinical data (n = 457) of pediatric gliomas were obtained from the CBTTC dataset (UCSC Xena) for model construction. Five independent datasets—Puget 2012 (n = 50, UCSC Xena), Cavalli (n = 538), Paugh (n = 42), Pomeroy (n = 57), and Witt (n = 23) from the Gliovis database—were used as validation cohorts to assess model stability and accuracy. All transcriptomic data were converted to TPM format and log2-transformed for subsequent analysis. To compare tumor samples with normal brain tissues, RNA-seq data (n = 207) from the GTEx database were used. Samples were filtered to include only patients under 18 years old with non-zero survival time. Additionally, gene expression and copy number variation (CNV) data from relevant cell lines were retrieved from CCLE (DepMap Data Downloads 23Q4). Batch effects across datasets were corrected using the Combat function in the sva package.

### 2.2 Acquisition and processing of scRNA-seq data

Single-cell RNA sequencing (scRNA-seq) data were obtained from the Gene Expression Omnibus (GEO) database under accession number GSE231859, comprising 19 pediatric glioma samples. Raw count matrices were processed in R (version 4.1.3) using the Seurat package. Quality control was performed to remove low-quality cells, potential doublets, and technical artifacts. Cells with fewer than 200 or more than 30,000 unique molecular identifiers (UMIs) were excluded to eliminate empty droplets and potential multiplets, respectively. Cells expressing fewer than 200 or more than 8,000 genes were removed to avoid low-complexity libraries and doublet contamination. To reduce the influence of stressed or dying cells, cells with mitochondrial gene expression exceeding 25% were filtered out, while cells with red blood cell gene expression greater than 3% were excluded to eliminate erythrocyte contamination.

Following quality control, gene expression data were normalized using the NormalizeData function with log-normalization, and highly variable genes were identified using FindVariableFeatures, selecting the top 2,000 genes based on variance-stabilizing transformation. The data were subsequently scaled using ScaleData. To minimize the confounding effects of cell cycle heterogeneity on downstream clustering and dimensionality reduction, cell cycle scores (S and G2M phase scores) were calculated and regressed out during data scaling.

Given that samples were derived from multiple patients and processed independently, batch effects were corrected using the Harmony algorithm, which aligns shared biological states across datasets while preserving biological variability. Harmony integration was performed based on the principal components derived from the highly variable genes, and convergence was assessed to ensure effective batch mixing across samples. The corrected embeddings were then used for downstream dimensionality reduction and clustering analyses.

Dimensionality reduction was conducted using both UMAP and t-distributed stochastic neighbor embedding (t-SNE) to visualize cellular heterogeneity. Cell clustering was performed using the Louvain algorithm implemented in Seurat, with resolution parameters optimized to balance cluster granularity and biological interpretability. Differential expression analysis between AIDPI-high and AIDPI-low tumor cells was performed using the FindAllMarkers function, with genes considered significant if they met the criteria of a nominal P value < 0.05, absolute log2 fold change > 0.25, and expression in at least 10% of cells within a cluster.

### 2.3 Cell annotation and CNV analysis

Cell annotation was conducted using established marker genes: glioma cells (SOX2, OLIG1, GFAP, S100B), oligodendrocytes (OLIG2, MBP), pericytes (ACTA2, PDGFRB), endothelial cells (PECAM1, CLDN5, FLT1, RAMP2), T cells (CD3D, CD3E, CD3G, TRAC), NK cells (NKG7, GNLY, NCAM1, KLRD1), B cells (CD79A, IGHM, IGHG3, IGHA2), myeloid cells (LYZ, MARCO, CD68, FCGR3A), and mast cells (KIT, MS4A2, GATA2). Cell annotation results were verified using scGate, followed by immune cell subtype refinement using sc-Type. To infer malignant epithelial cells, inferCNV was applied, using endothelial and fibroblast cells as a reference.

### 2.4 Identification of prognostic genes

To systematically identify genes associated with overall survival in pediatric gliomas, univariate Cox proportional hazards regression was applied to genome-wide expression profiles in the CBTTC cohort, with overall survival time and event status as outcome variables. To ensure that identified prognostic signals were not driven by cohort-specific effects or random variation inherent to small pediatric datasets, the same Cox regression procedure was independently performed in five external cohorts, including Puget 2012, Cavalli, Paugh, Pomeroy, and Witt. Genes were retained only if they reached statistical significance (P < 0.05) in at least five datasets, thereby enforcing cross-cohort reproducibility as a prerequisite for downstream modeling. This strategy substantially reduced dimensionality while preserving prognostic stability across heterogeneous platforms and clinical backgrounds.

### 2.5 Differential gene and enrichment analysis

Differential gene expression analysis was conducted using the limma package, comparing high- and low-AIDPI groups. Genes with p < 0.05 and |log2FC| > 0.2 were considered significantly differentially expressed. Functional enrichment analysis was performed using clusterProfiler with GSEA, leveraging HALLMARK and KEGG gene sets from MSigDB. Enrichment results were visualized using the enrichplot package.

### 2.6 Development of a tumor-associated risk signature

A prognostic model was developed using 101 machine learning algorithms, assigning risk scores to individual patients. Patients were categorized into high-risk and low-risk groups based on an optimal cutoff value determined by the surv_cutpoint function. The predictive performance of the model was evaluated across validation cohorts.

### 2.7 Machine learning-based integrated risk prediction model

To avoid reliance on a single modeling assumption and to address the high-dimensional, low-sample-size nature of pediatric glioma datasets, a comprehensive artificial intelligence–based modeling framework was implemented, integrating linear, nonlinear, tree-based, and kernel-based survival learning algorithms. Penalized regression approaches, including Lasso, Ridge, and Elastic Net, were used to handle multicollinearity and reduce overfitting by shrinking regression coefficients. Tree-based ensemble methods such as Random Survival Forest and gradient boosting models captured nonlinear effects and higher-order interactions, while CoxBoost and plsRcox addressed feature redundancy through iterative boosting and latent component extraction. Survival support vector machines were applied to model complex decision boundaries in the feature space.

All models were trained in the CBTTC cohort using leave-one-out cross-validation, a strategy specifically chosen to maximize training data utilization while providing nearly unbiased performance estimates in small cohorts. Model generalizability was rigorously evaluated in five independent validation datasets, and prognostic performance was quantified using Harrell’s concordance index. The final AIDPI model was selected based on the highest average C-index across all cohorts, reflecting both predictive accuracy and robustness. This multi-algorithm comparison and multi-cohort validation framework effectively minimized overfitting and ensured the reproducibility of the final risk signature.

### 2.8 Strategies for overfitting control and model robustness

Given the inherent challenges of pediatric oncology datasets, several complementary strategies were implemented to prevent overfitting. Prognostic gene selection required consistency across multiple independent cohorts rather than reliance on a single dataset. Model training incorporated regularization, boosting, and cross-validation techniques to constrain model complexity. Most importantly, all candidate models were evaluated in external cohorts that were not involved in model training, ensuring that the reported performance reflects true generalizability rather than internal optimization.

### 2.9 Tumor immune infiltration analysis

Immune infiltration levels in CBTTC patients were quantified using the IOBR package with the ESTIMATE algorithm. This approach provided estimates of stromal, immune, and tumor cell fractions within the tumor microenvironment. A heatmap was generated to compare immune infiltration patterns between high- and low-risk groups.

### 2.10 cell culture

Human glioma cell lines U87, U251, U-118 MG, LN-18 and T98G were obtained from the American Type Culture Collection (ATCC, Manassas, VA, USA), while normal human astrocytes (NHA) were purchased from ScienCell Research Laboratories (Carlsbad, CA, USA). All cells were cultured in high-glucose Dulbecco’s Modified Eagle Medium (DMEM; Solarbio, Beijing, China) supplemented with 10% fetal bovine serum (FBS) and 1% penicillin-streptomycin. Cells were maintained at 37 °C in a humidified atmosphere containing 5% CO_2_.

### 2.11 RNA extraction and quantitative real-time PCR

Total RNA was extracted using TRIzol reagent (Invitrogen, Carlsbad, CA, USA) following the manufacturer’s protocol. Complementary DNA (cDNA) was synthesized from 1 µg of total RNA using the ReverTra Ace qPCR RT Master Mix with gDNA Remover (Toyobo, Japan). Quantitative real-time PCR was carried out using SYBR Premix Ex Taq II (Takara, Japan) on an Mx3005P real-time PCR system (Agilent Technologies, Santa Clara, CA, USA). GAPDH was used as the internal control. The thermal cycling conditions were as follows: initial denaturation at 95 °C for 10 minutes, followed by 45 cycles of denaturation at 95 °C for 5 seconds and annealing/extension at 60 °C for 30 seconds. Each reaction was performed in triplicate. Gene-specific primers were used for amplification, and relative gene expression levels were calculated using the 2^(-ΔΔCt) method. Primer sequences are listed in **Supplementary File 1**.

### 2.121 siRNA-mediated gene silencing of PRKAR2A

U-118 MG and LN-18 glioma cells (2×10^5^ cells per well) were transfected with 50 nM siRNA targeting PRKAR2A or a non-targeting control siRNA (NC) using Lipofectamine 3000 (Invitrogen) in Opti-MEM medium. After 6 hours of transfection, the medium was replaced with complete DMEM supplemented with 10% fetal bovine serum (FBS). At 48 hours post-transfection, the efficiency of PRKAR2A knockdown (>70%, p < 0.01 vs. NC) was verified by quantitative real-time PCR (qRT-PCR).

### 2.13 Cell proliferation assay (CCK-8)

Cells were seeded in 96-well plates at a density of 3×10³ cells per well and transfected as described above. At 24, 48, 72, and 96 hours post-transfection, 10 µL of CCK-8 reagent (Dojindo, Japan) was added to each well, followed by incubation at 37 °C for 2 hours. Absorbance was measured at 450 nm using a microplate reader (BioTek Synergy H1). Each experimental group was performed in quintuplicate, and cell proliferation was quantified as fold change relative to the baseline measurement at the 0-hour time point.

### 2.14 Migration and invasion assays

Migration Assay: A total of 5×10^4^ transfected cells were seeded in serum-free medium in the upper chamber of Transwell inserts (8-µm pores; Corning, USA). The lower chamber was filled with complete medium containing 10% FBS to act as a chemoattractant. After 24 hours of incubation, non-migrated cells on the upper surface of the membrane were gently removed with a cotton swab. Migrated cells on the lower surface were fixed with 4% paraformaldehyde (PFA), stained with 0.1% crystal violet, and then imaged using a light microscope. Cell counts were obtained from five randomly selected fields per well.

Invasion Assay: For invasion assays, Transwell inserts were pre-coated with Matrigel (1:8 dilution in serum-free DMEM; BD Biosciences) and allowed to polymerize at 37 °C for 4 hours. Next, 5×10^4^ transfected cells in serum-free medium were seeded into the upper chamber, while the lower chamber contained complete medium with 10% FBS. After 24 hours of incubation, non-invading cells were removed, and invaded cells were fixed, stained, and quantified following the same protocol as for the migration assay.

### 2.15 Western blotting

Proteins were extracted from transfected cells using RIPA buffer (Beyotime) supplemented with protease inhibitors (Roche). Protein concentrations were determined using the BCA protein assay (Pierce). Equal amounts of protein (30 µg) were separated by 10% SDS-PAGE and transferred to PVDF membranes (Millipore). The membranes were incubated overnight at 4 °C with primary antibodies against PRKAR2A (1:1000; Abcam) and β-actin (1:1000; Santa Cruz Biotechnology). Following washing, membranes were incubated with HRP-conjugated secondary antibodies (1:5000; Sigma-Aldrich) for 1 hour at room temperature. Protein bands were visualized using SuperSignal™ West Pico Chemiluminescent Substrate (Thermo Fisher) and imaged using the ChemiDoc™ MP Imaging System (Bio-Rad). Band intensities were quantified using ImageJ software (National Institutes of Health, Bethesda, MD, USA).

### 2.16 Statistical analysis

All statistical analyses and visualizations were performed using R (v4.1.3). Correlations between continuous variables were assessed using Pearson correlation coefficients. Chi-square tests were used for categorical variables, while Wilcoxon rank-sum tests or t-tests were applied to compare continuous variables. Optimal cutoff values for survival analysis were determined using the survminer package. Kaplan-Meier survival curves and Cox regression models were generated using the survival package.

## 3 Results

### 3.1 AIDPI model demonstrates superior prognostic performance in pediatric gliomas

**Figure 1:**
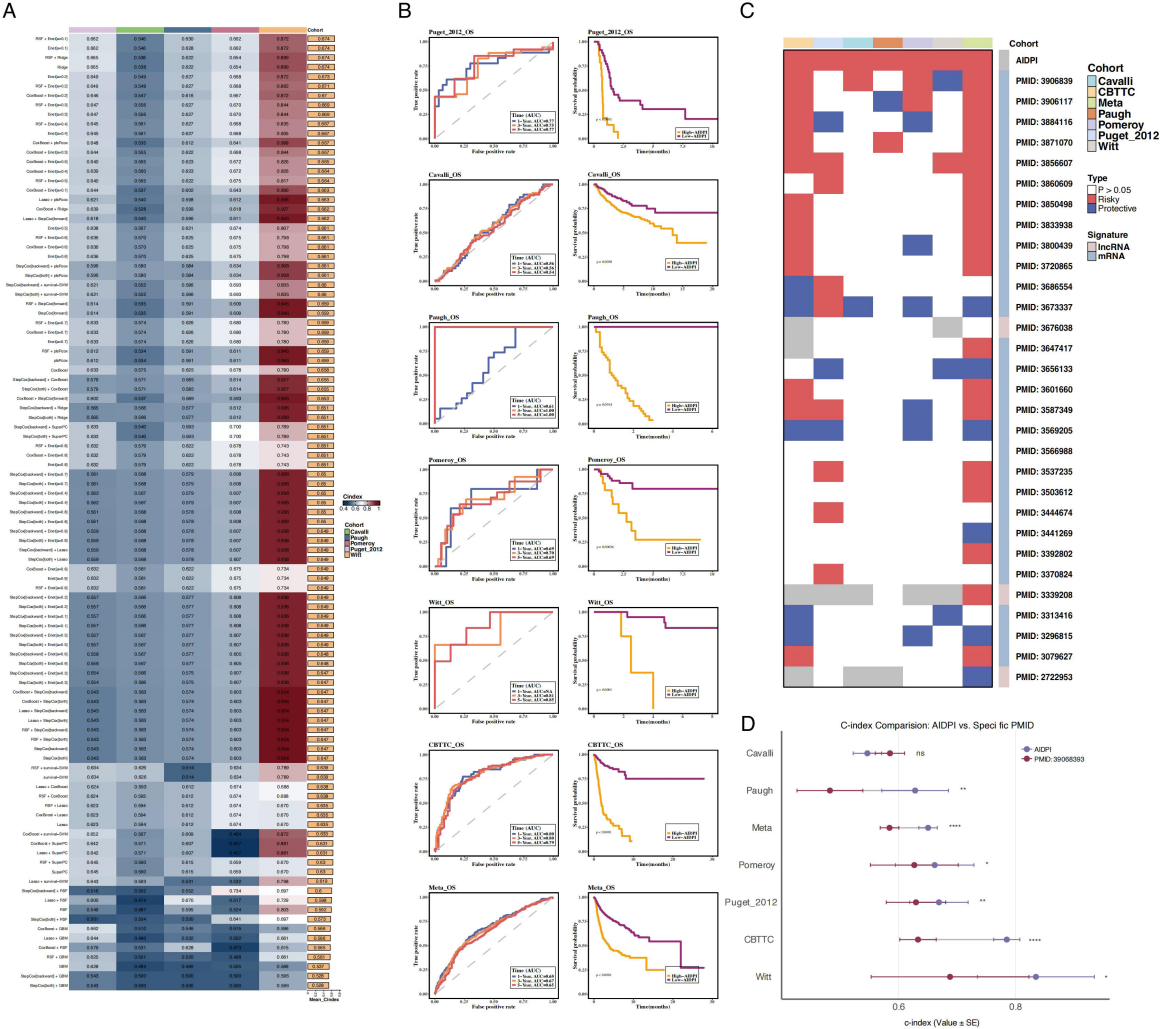
Performance evaluation of the AIDPI model across multiple datasets. **(A)** Heatmap of C-index values for 101 prognostic models across multiple datasets, with red and blue indicating high and low C-index values, respectively. **(B)** Time-dependent ROC curves for 1-, 3-, and 5-year survival predictions (7 ROC curves shown) and Kaplan -Meier survival analyses across various datasets. **(C)** Heatmap comparing AIDPI with 32 recently published glioma prognostic models, highlighting significant differences. **(D)** Bar plot comparing C-index values of AIDPI with the top-performing models from 30 prognostic studies across seven datasets.

Using gene expression profiles from the CBTTC dataset and five independent validation cohorts (Puget 2012, Cavalli, Paugh, Pomeroy, and Witt), we performed univariate Cox regression analysis to identify genes associated with prognosis in pediatric gliomas. Nine genes (GRIA1, ZNF165, TM9SF2, PRKAR2A, PSMD6, H1F0, CDC25B, HIST1H2AE, and NCAPD2) were consistently significant across at least five datasets (P < 0.05) (**Supplementary Figure 1A**). We then incorporated these genes into an AIDPI (Artificial Intelligence-Derived Prognostic Index) model and evaluated 101 machine learning algorithms based on C-index performance. The Enet model [α = 0.1] was selected as the optimal model due to its superior C-index across all datasets (**Figure 1A**, **Supplementary Figures 1B, C**).

The AIDPI model was subsequently validated across seven datasets using time-dependent ROC curves and Kaplan–Meier survival analysis, both of which consistently demonstrated that patients in the high-AIDPI group exhibited significantly poorer survival outcomes (**Figure 1B**, **Supplementary Figures 1D, E**). Further, we compared AIDPI with 30 recently published glioma prognostic models, revealing that AIDPI outperformed these models (**Figure 1C**). Additionally, when benchmarking against the top-performing models from these studies, AIDPI demonstrated superior C-index values across most datasets (**Figure 1D**).

### 3.2 AIDPI is an independent risk factor for survival

**Figure 2:**
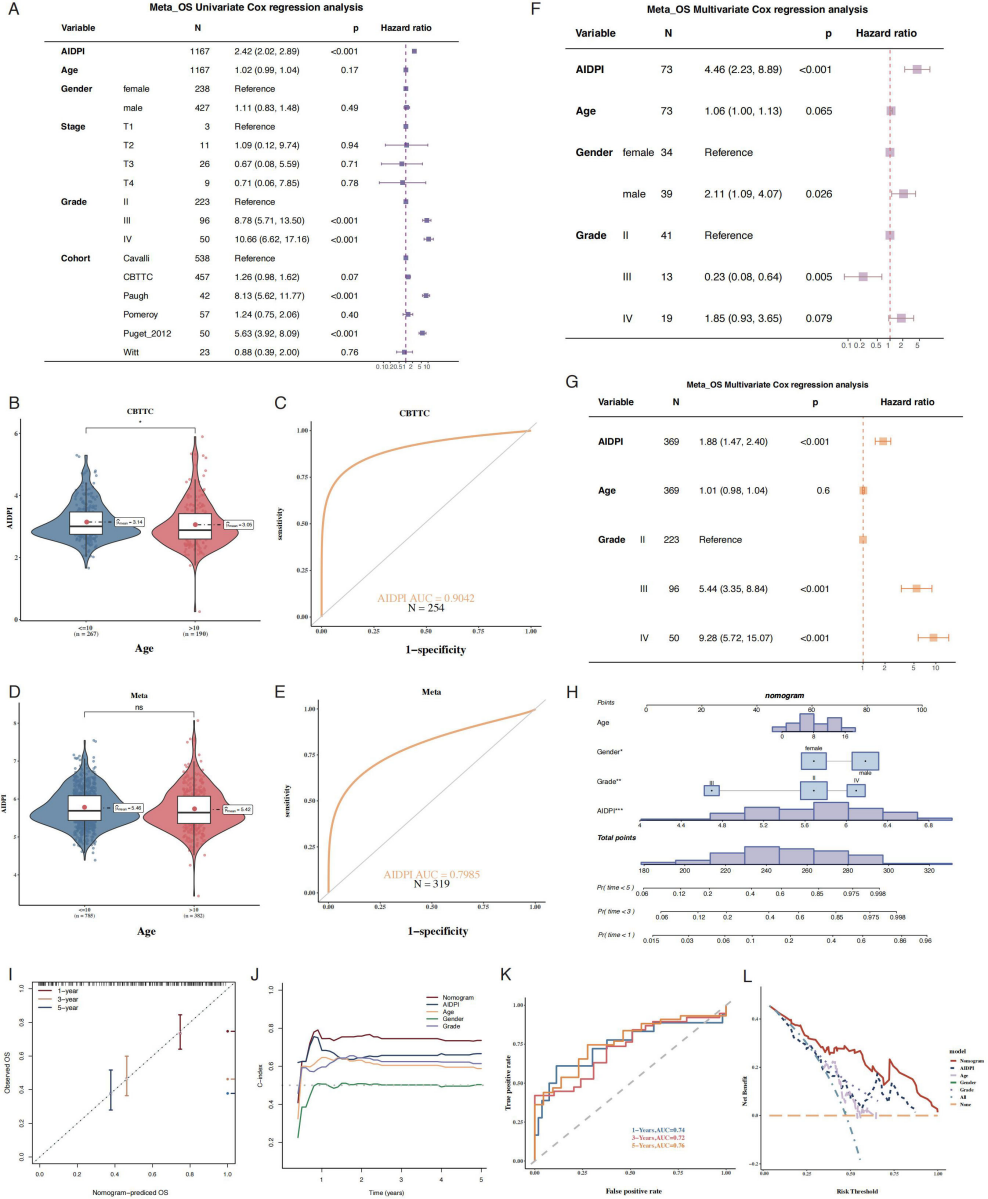
AIDPI as an independent prognostic factor. **(A)** Forest plot summarizing univariate Cox regression analysis results for AIDPI, age, gender, stage, grade, and cohort in the Meta dataset. **(B–E)** Chemotherapy response prediction based on AIDPI in the CBTTC and Meta datasets. **(F–G)** Forest plots for multivariate Cox regression analysis incorporating AIDPI, age, gender, grade, and cohort. **(H)** Nomogram integrating AIDPI, age, and grade to predict patient survival. **(I–L)** Model validation using calibration curves, time-dependent ROC, time-dependent C-index, and decision curve analysis (DCA).

We conducted univariate Cox regression analysis incorporating AIDPI, age, gender, stage, grade, and cohort, identifying AIDPI as an independent risk factor (**Figure 2A**). Further analysis in the CBTTC and Meta cohorts revealed that AIDPI effectively distinguished age groups, with patients aged ≤10 years showing higher AIDPI scores (**Figures 2B–E**). **Supplementary Figures 2A–C** shows violin plots of AIDPI values across different tumor grades, gender, and stage, where Grade III tumors exhibited the highest AIDPI values, gender showed no significant differences, and T4 tumors had the lowest AIDPI values.

Multivariate Cox analysis adjusting for age, gender, and grade confirmed that AIDPI remained a significant prognostic indicator (**Figures 2F, G**). **Supplementary Figures 2D–F** presents the forest plots of Cox analysis for AIDPI values in relation to tumor grade, age, and gender, highlighting its independent prognostic value. Based on these findings, we constructed a nomogram integrating AIDPI, age, and grade to predict patient survival (**Figure 2H**). Model performance was further validated using calibration curves, time-dependent ROC, time-dependent C-index, and decision curve analysis (DCA), all of which indicated high predictive accuracy (**Figures 2I–L**).

### 3.3 High AIDPI scores indicate immune suppression and cell adhesion alterations

**Figure 3:**
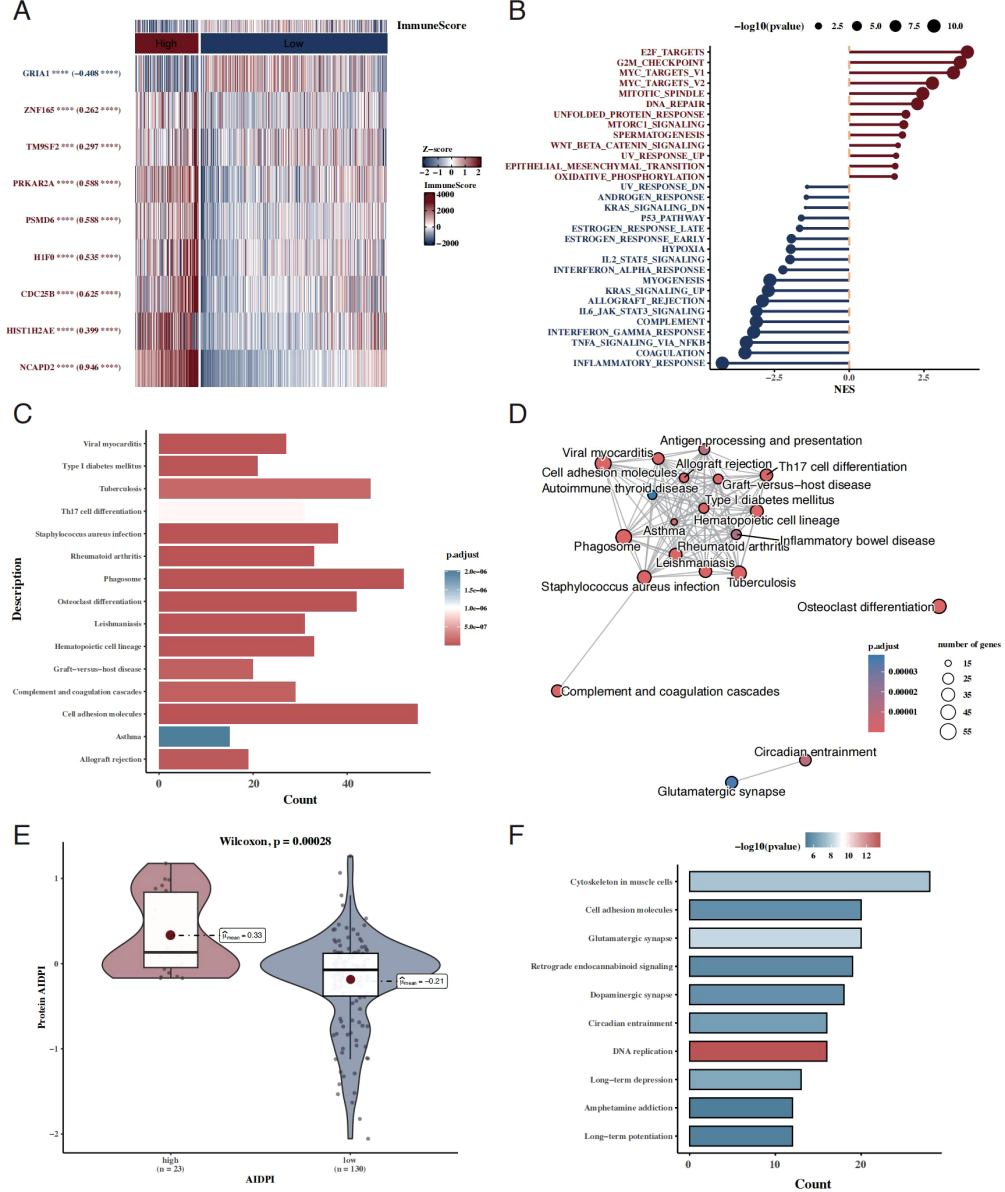
Functional characterization of AIDPI-associated genes and pathways. **(A)** Heatmap showing differential expression of the nine AIDPI model genes between high- and low-AIDPI groups. **(B)** Lollipop plot displaying GSEA results for differentially expressed genes (DEGs) between the two groups. **(C, D)** Bubble plot and network diagram of overrepresentation analysis (ORA) for AIDPI-associated DEGs. **(E)** Violin plot comparing AIDPI values between groups based on proteomics data. **(F)** Bar chart of KEGG pathway enrichment analysis for differentially expressed proteins.

We examined the expression differences of the nine AIDPI model genes between the high- and low-AIDPI groups and assessed their immune scores, revealing that patients in the high-AIDPI group exhibited lower immune scores (**Figure 3A**). Differentially expressed genes (DEGs) between the two groups were identified using limma, followed by GSEA pathway analysis, which highlighted significant upregulation of E2F targets and downregulation of inflammatory pathways in the high-AIDPI group (**Figure 3B**).

Further, KEGG enrichment analysis and functional network analysis using ORA suggested that AIDPI-associated DEGs were involved in pathways related to cell adhesion and autophagy (**Figures 3C, D**). Protein-level validation using proteomics data demonstrated significant differences in AIDPI values between groups (**Figure 3E**). A follow-up KEGG enrichment analysis reaffirmed the involvement of cell adhesion pathways (**Figure 3F**).

### 3.4 Single-cell analysis identifies three key prognostic genes from the AIDPI model

**Figure 4:**
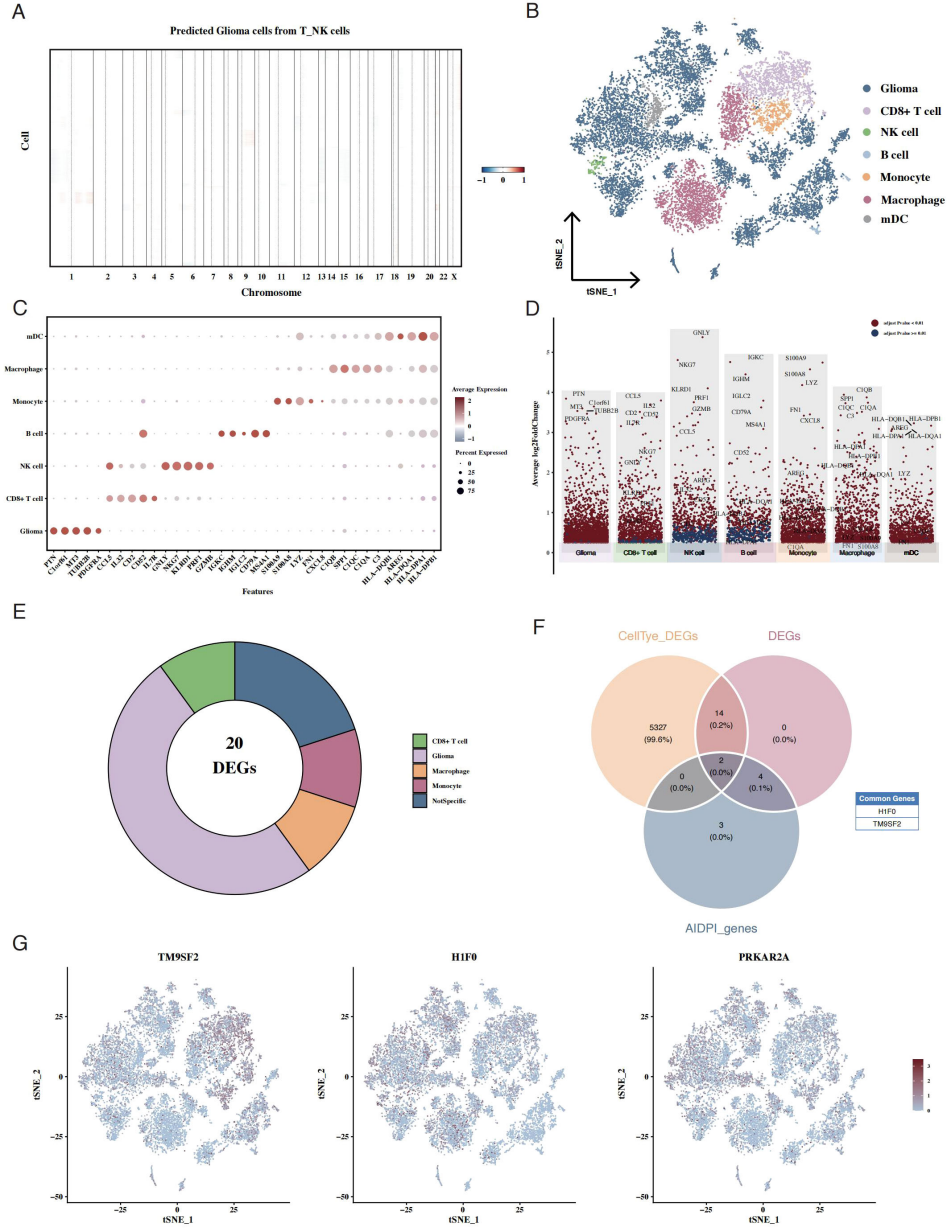
Single-cell analysis identifies key prognostic genes from the AIDPI model. **(A)** CNV heatmap inferred using inferCNV, showing chromosomal alterations in glioma cells. **(B)** tSNE plot depicting cell-type annotations in single-cell data. **(C)** Bubble plot illustrating marker gene expression across different cell types. **(D)** Volcano plot showing differentially expressed marker genes across cell types. **(E)** Pie chart representing marker gene expression in high- and low-AIDPI tumor cells. **(F)** Venn diagram displaying overlapping genes among cell-type markers, AIDPI-associated DEGs, and AIDPI model genes. **(G)** tSNE plots visualizing the expression of TM9SF2, H1F0, and PRKAR2A in glioma cells.

Using the inferCNV package, we inferred malignant cells within the epithelial compartment, using T/NK cells as a reference (**Figure 4A**). To enhance cell type annotation, we employed the scGate algorithm with the UCell scoring method to generate tSNE plots depicting glioma, stromal, immune, and lymphocyte scores (**Supplementary Figure 3A**). Additionally, scGate-based classification using CD45 expression effectively distinguished immune and non-immune cells (**Supplementary Figure 3B**). To further delineate malignant and normal cells within the glioma population, we applied inferCNV to predict CNV signals using T/NK cells as a reference, generating CNV signal scatter plots to differentiate malignant from normal glioma cells (**Supplementary Figure 3C**). Corresponding CNV heatmaps for T/NK reference cells and inferred normal glioma cells further validated the classification (**Supplementary Figures 3D, E**). Cell annotation based on canonical marker genes identified seven distinct cell populations, including glioma cells, macrophages, monocytes, mDCs, B cells, NK cells, and CD8+ T cells (**Figures 4B–D**). Using a resolution of 0.8, we performed cell clustering analysis, as illustrated in the tSNE plot (**Supplementary Figure 3F**). The expression of key lineage markers further confirmed the annotation: glioma cell marker SOX2, T cell marker CD3D, and myeloid cell marker CD68 showed distinct expression patterns across clusters (**Supplementary Figures 3G–I**). Furthermore, we visualized cell-type distributions for each patient using tSNE plots (**Supplementary Figure 3J**). We then applied AUCell scoring to classify high- and low-AIDPI tumor cells, subsequently performing differential expression analysis between the two groups (**Figure 4E**). By integrating cell type markers, AIDPI-associated DEGs, and AIDPI model genes, we identified two key overlapping genes: TM9SF2 and H1F0 (**Figure 4F**). Additionally, we visualized the expression of TM9SF2, H1F0, and PRKAR2A across glioma cells (**Figure 4G**).

### 3.5 High H1F0 expression predicts poor prognosis in pediatric gliomas

**Figure 5:**
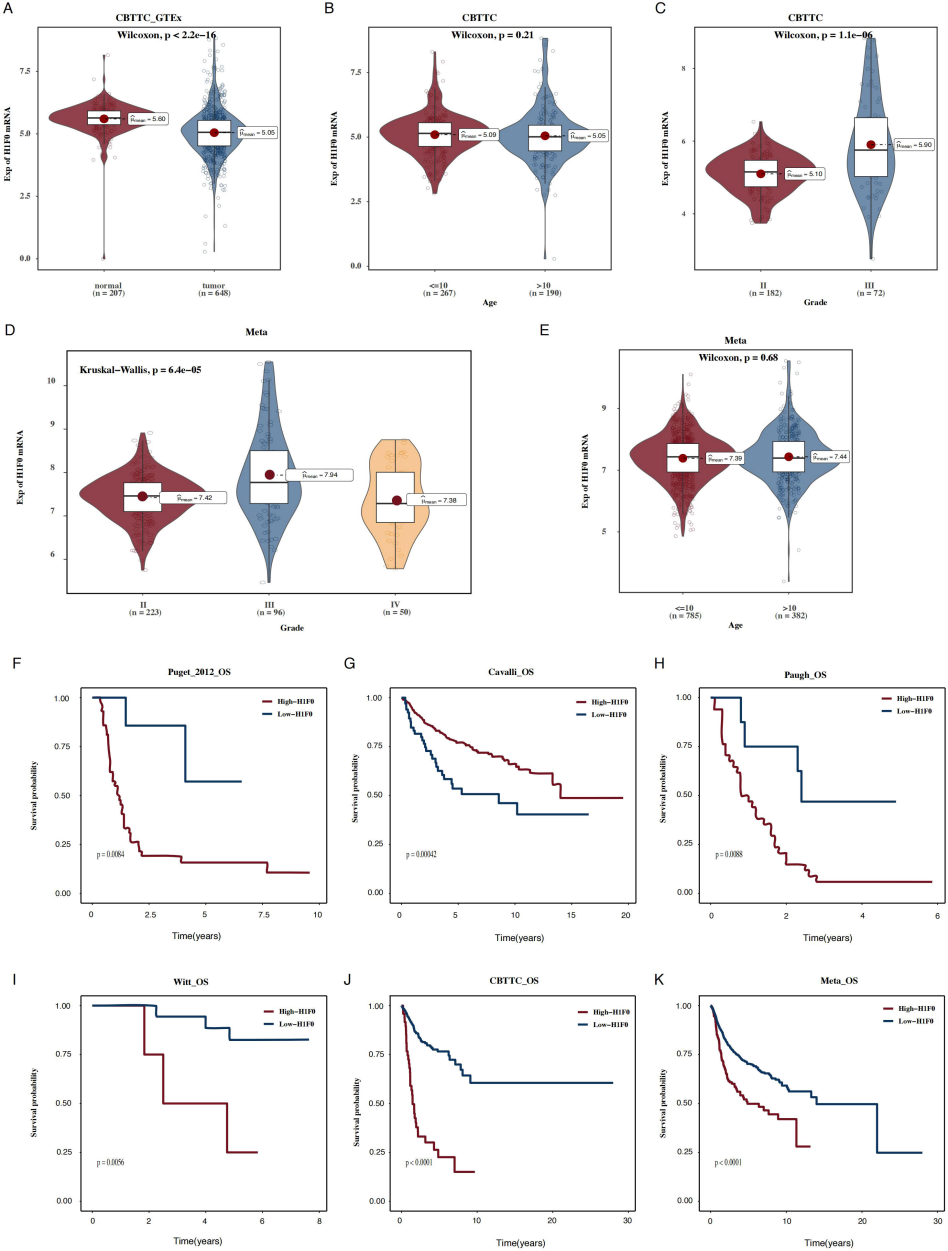
H1F0 expression and prognostic relevance in pediatric gliomas. **(A)** Violin plot showing differential H1F0 expression between normal and tumor tissues. **(B, C)** Box plots illustrating H1F0 expression across different age groups and tumor grades in the CBTTC dataset. **(D, E)** Box plots depicting H1F0 expression across different age groups and tumor grades in the Meta dataset. **(F–K)** Kaplan–Meier survival curves across six independent datasets.

From the copy number variation (CNV) analysis and expression patterns of H1F0 across different tumor types (**Supplementary Figures 4A, B**), we observed that H1F0 exhibited significant CNV alterations and distinct expression patterns in gliomas. We further explored its expression dynamics and prognostic relevance. H1F0 expression was markedly reduced in tumor tissues (**Figure 5A**), with no significant association with patient age (**Figure 5B**). Interestingly, H1F0 expression varied across tumor stages, with higher levels observed in Grade III tumors (**Figures 5C–E**). Finally, across six independent datasets, higher H1F0 expression was consistently associated with poor patient prognosis (**Figures 5F–K**).

**Figure 6:**
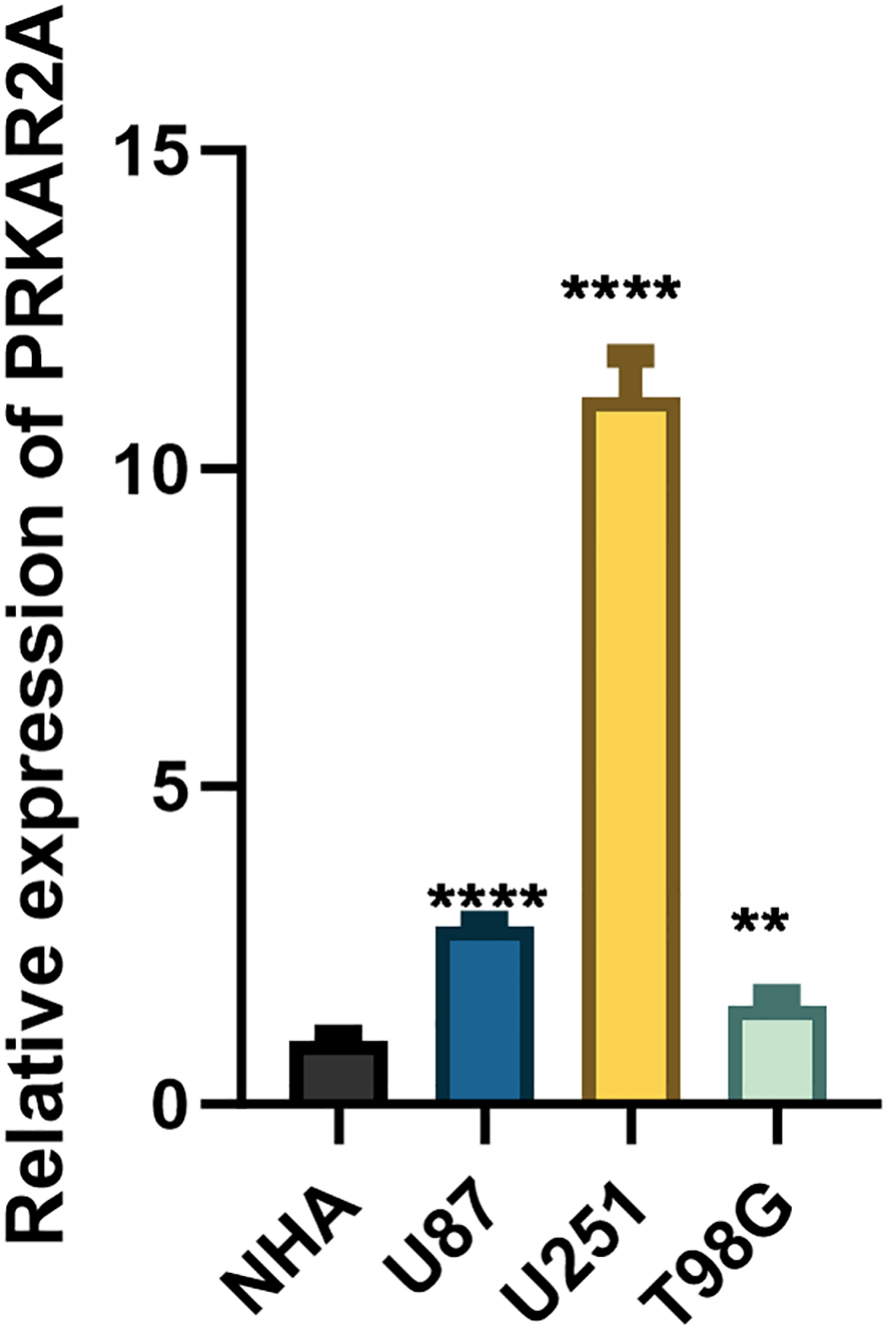
qRT-PCR validation of PRKAR2A expression in glioma cells. Bar graph showing the relative mRNA expression levels of PRKAR2A in glioma cell lines (U87, U251, and T98G) compared to normal human astrocytes (NHA), as determined by quantitative real-time PCR. (** for p < 0.01, and **** for p < 0.0001).

To validate the AIDPI model at the transcript level, we performed quantitative real-time PCR (qRT-PCR) analysis of PRKAR2A expression in glioma cell lines (U87, U251, and T98G) and normal human astrocytes (NHA). The results confirmed that PRKAR2A expression was significantly upregulated in all glioma cell lines compared to normal astrocytes (**Figure 6**), further supporting its role as a potential oncogenic factor in pediatric gliomas.

### 3.6 PRKAR2A knockdown inhibits glioma cell proliferation, migration, and invasion

**Figure 7:**
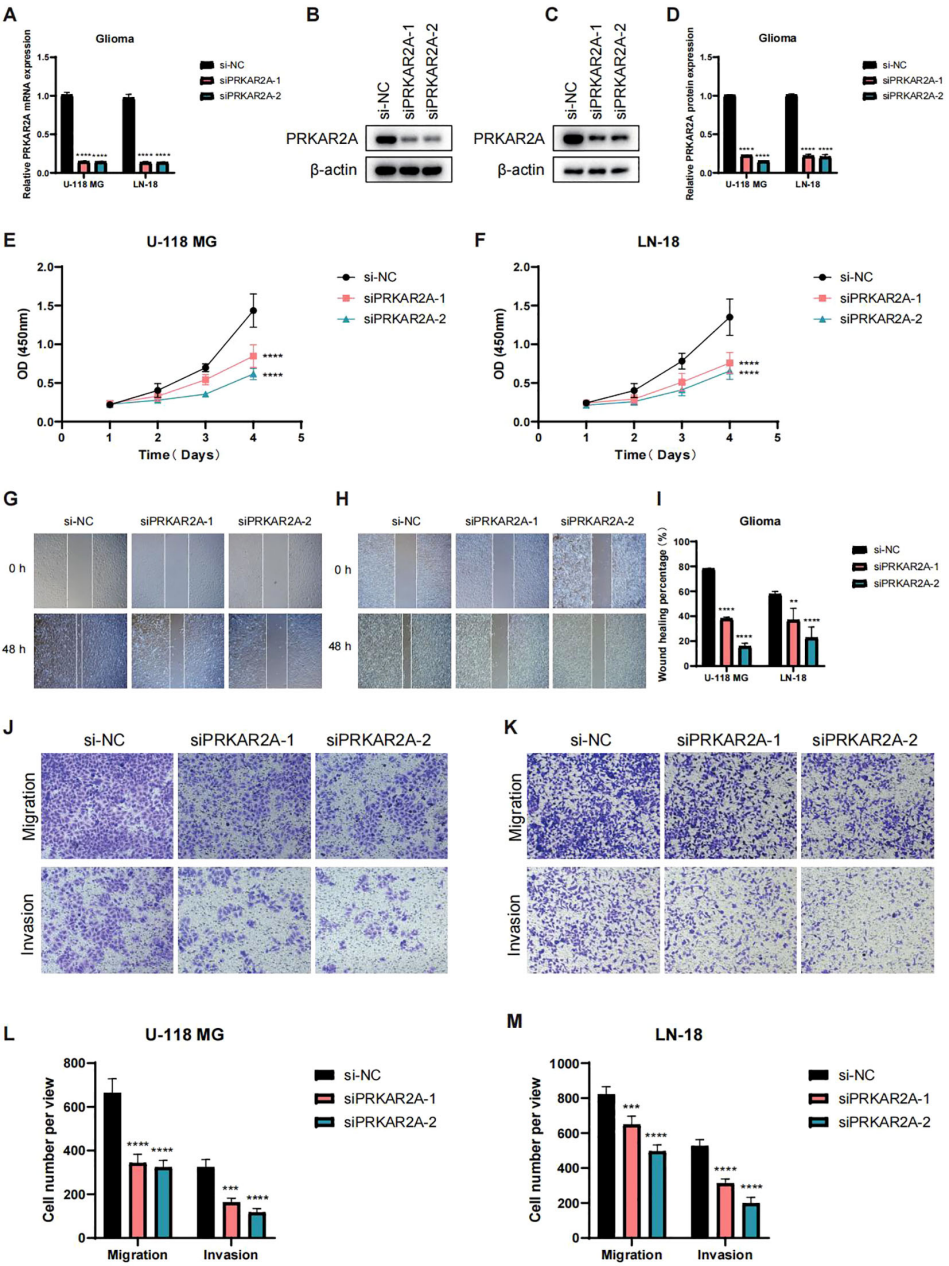
Functional validation of PRKAR2A knockdown in glioma cells. **(A)** Bar graph showing the relative mRNA expression levels of PRKAR2A in U-118 MG and LN-18 glioma cells following knockdown with two different siRNAs, as measured by quantitative real-time PCR (qRT-PCR). **(B–D)** Western blot (WB) analysis validating the efficient knockdown of PRKAR2A in U-118 MG cells and LN-18 cells. **(E)** Cell proliferation curves for U-118 MG wild-type and PRKAR2A knockdown cells, demonstrating significantly reduced cell growth following knockdown. **(F)** Cell proliferation curves (OD 450) for LN-18 wild-type and PRKAR2A knockdown cells, showing a significant decrease in cell growth after knockdown. **(G–I)** Wound healing assay showing significantly impaired migration of U-118 MG cells and LN-18 cells after PRKAR2A knockdown. **(J–M)** Migration and invasion assays showing significant reduction in both migration and invasion of U-118 MG cells and LN-18 cells after PRKAR2A knockdown. (** for p < 0.01, *** for p < 0.001, and **** for p < 0.0001).

To further investigate the functional role of PRKAR2A in glioma cells, we performed a series of *in vitro* experiments using siRNA-mediated knockdown of PRKAR2A in U-118 MG and LN-18 glioma cell lines. qRT-PCR analysis confirmed efficient knockdown of PRKAR2A in both cell lines (**Figure 7A**). WB analysis further validated the reduced PRKAR2A protein levels in both U-118 MG and LN-18 cells (**Figures 7B–D**). Next, we assessed the impact of PRKAR2A knockdown on cell proliferation by measuring the optical density (OD 450) of U-118 MG and LN-18 cells. Both cell lines showed significantly inhibited proliferation following PRKAR2A knockdown (**Figures 7E, F**). These findings suggest that PRKAR2A plays a key role in promoting cell growth in gliomas. Wound healing assays were performed to assess the effect of PRKAR2A knockdown on cell migration. Results showed that migration was significantly impaired in both U-118 MG and LN-18 cells upon PRKAR2A knockdown (**Figures 7G–I**). Finally, we evaluated the effects of PRKAR2A knockdown on glioma cell invasion and migration using transwell assays. Both U-118 MG and LN-18 cells exhibited significantly reduced invasion and migration following PRKAR2A knockdown (**Figures 7J–M**). Together, these results suggest that PRKAR2A contributes to the proliferation, migration, and invasion of glioma cells, highlighting its potential as a therapeutic target in pediatric gliomas.

## 4 Discussion

In this study, we integrated transcriptomic and single-cell sequencing data to develop the AIDPI model, an AI-driven prognostic index for pediatric gliomas. We identified nine key survival-associated genes and optimized an Enet-based model, which outperformed existing prognostic tools. AIDPI was validated as an independent risk factor and effectively stratified tumor grades. Single-cell analysis highlighted TM9SF2 and H1F0 in malignant glioma cells, with H1F0 overexpression linked to poor prognosis. This study establishes a robust AI-based prognostic tool and reveals novel molecular targets in pediatric gliomas.

The integration of artificial intelligence (AI) in prognostic modeling has revolutionized outcome prediction across various medical fields, particularly in oncology. In this study, we developed and validated the AIDPI model, an AI-driven prognostic tool for pediatric gliomas, demonstrating superior predictive performance compared to traditional models. Using a rigorous feature selection process, we identified nine key prognostic genes (GRIA1, ZNF165, TM9SF2, PRKAR2A, PSMD6, H1F0, CDC25B, HIST1H2AE, and NCAPD2), which were consistently associated with survival across multiple cohorts. By evaluating 101 machine learning algorithms, we determined that the Enet model (α = 0.1) provided optimal performance, achieving the highest C-index values across six independent datasets. The prognostic superiority of AI-based models has been extensively documented in various cancers. For instance, an AIDPI model developed for osteosarcoma demonstrated improved survival prediction over traditional clinicopathological factors, facilitating high-risk patient stratification and guiding treatment decisions (10). Similarly, AI-driven prognostic signatures in hepatocellular carcinoma have outperformed conventional methods by incorporating transcriptomic and biological markers, improving the accuracy of survival predictions (11, 12). Beyond oncology, AI-based models in cardiovascular diseases have enhanced risk assessment in acute heart failure and transcatheter aortic valve replacement, surpassing traditional scoring systems (13, 14). These findings underscore the transformative role of AI in clinical decision-making.

Pediatric gliomas exhibit a complex and heterogeneous tumor microenvironment, characterized by profound immune suppression and dysregulated cell adhesion, both of which contribute to tumor progression and therapeutic resistance (15). Our study revealed that high AIDPI scores are associated with lower immune scores, suggesting an immunosuppressive microenvironment. This aligns with previous findings that pediatric gliomas exploit immune checkpoint molecules, such as PD-L1, to suppress anti-tumor immune responses by promoting myeloid-derived suppressor cells (MDSCs) and tumor-associated macrophages (TAMs), both of which inhibit T-cell proliferation and function (16). GSEA results further supports an immune-cold phenotype in high-AIDPI tumors, showing upregulation of E2F target genes and downregulation of inflammatory pathways. These results suggest that high-risk gliomas may evade immune surveillance through glycan-mediated inhibitory signaling, which is known to modulate immune cell activity in the glioma microenvironment (17). Such immunosuppressive adaptations not only limit the effectiveness of immune checkpoint blockade therapies but also contribute to tumor persistence and recurrence. Beyond immune suppression, KEGG pathway analysis implicated AIDPI-related genes in cell adhesion and autophagy, further corroborated by proteomic data. Notably, N-cadherin, a key adhesion molecule, has been reported to be highly expressed in pediatric gliomas and linked to shorter survival times by facilitating glioma cell migration and interaction with neurons and astrocytes (18). This dynamic regulation of cell adhesion pathways allows tumor cells to adapt to different microenvironments, enhancing invasiveness and metastatic potential. Targeting these pathways holds therapeutic promise. Recent studies suggest that MAPKAPK2 inhibition can suppress glioma proliferation and migration while altering immune cell infiltration (19). Similarly, the PI3K/AKT/mTOR pathway, which plays a role in epigenetic reprogramming of immune cells, has emerged as a potential therapeutic target to overcome glioma resistance to conventional therapies (20). Taken together, our findings suggest that high-AIDPI tumors exhibit a dual oncogenic strategy-immune evasion and enhanced adhesion-both of which contribute to tumor progression. Understanding these mechanisms provides new therapeutic avenues for targeting immune suppression and adhesion pathways in pediatric gliomas, potentially improving treatment outcomes.

H1F0, a histone linker protein involved in chromatin remodeling, exhibits significant copy number variations (CNVs) and altered expression patterns in pediatric gliomas. Although H1F0 expression is generally reduced in tumor tissues, we observed a paradoxical increase in Grade III tumors, suggesting a context-dependent role in glioma progression. This pattern aligns with studies in other cancers, where H1F0 regulation is tightly linked to tumor cell proliferation and senescence. For instance, in bladder cancer, HINFP knockout transcriptionally inhibited H1F0 and H1FX, leading to DNA damage and cellular senescence, ultimately suppressing tumor growth (21). This suggests that H1F0 may be involved in chromatin integrity maintenance, with its dysregulation contributing to tumor progression. More importantly, higher H1F0 expression correlated with poor prognosis across six independent datasets, further underscoring its potential role as a biomarker for aggressive gliomas. Previous reports have also linked H1F0 expression to stromal and immune scores in lung adenocarcinoma (LUAD), indicating a broader impact of chromatin remodeling on the tumor microenvironment (22). These findings suggest that H1F0 may influence not only tumor cell proliferation but also immune regulation, potentially shaping the glioma microenvironment. Given its dual role in chromatin organization and gene regulation, H1F0 represents a promising biomarker for glioma progression and an attractive therapeutic target for high-risk patients. Future studies focusing on the functional consequences of H1F0 dysregulation in gliomas could provide insights into its mechanistic role and therapeutic potential in pediatric brain tumors.

In addition to H1F0, TM9SF2 and PRKAR2A play pivotal roles in the pathogenesis of pediatric gliomas. TM9SF2, implicated in pancreatic cancer (23), regulates metabolic pathways and cell adhesion, processes that are key to glioma invasion. Its regulation of lactate dehydrogenase A (LDH-A) suggests a role in metabolic reprogramming (24), which supports the rapid proliferation and aggressiveness of glioma cells. Similarly, PRKAR2A, a tumor suppressor involved in PKA signaling, has been shown to regulate cell cycle and apoptosis. Loss of PRKAR2A, as observed in fibrolamellar carcinoma (25), could drive glioma progression by enhancing mRNA stability and promoting an undifferentiated, proliferative state. Together, these genes contribute to glioma malignancy by modulating key processes such as cell adhesion, metabolism, and differentiation, highlighting their potential as therapeutic targets and their inclusion in the AIDPI model for better patient risk stratification.

While this study establishes a robust AI-derived prognostic model with significant predictive power and cross-dataset validation, certain limitations should be noted. The retrospective design and reliance on public transcriptomic datasets may restrict the generalizability and clinical applicability of the AIDPI model. Future prospective, multi-center studies are needed to further evaluate its real-world utility and robustness. Moreover, although the model associates a high AIDPI score with an immunosuppressive tumor microenvironment, the specific mechanisms by which the nine key genes—particularly those beyond PRKAR2A—contribute to immune evasion or dysregulated cell adhesion remain underexplored. Future work should integrate functional genomics, spatial transcriptomics, and immune profiling to dissect the molecular and cellular pathways underlying this immunosuppressive phenotype. Experimental validation of additional candidate genes such as H1F0 and TM9SF2 in relevant glioma models would further clarify their roles in tumor progression and therapy resistance. Such efforts will be crucial for translating this prognostic signature into actionable therapeutic strategies in pediatric neuro-oncology.

## 5 Conclusion

In conclusion, our study highlights AIDPI as a powerful prognostic tool for pediatric gliomas, with potential implications for risk stratification and targeted therapy development. Future efforts should focus on validating AIDPI in clinical settings and exploring its role in guiding precision oncology approaches for pediatric glioma patients.

## Data Availability

The datasets presented in this study can be found in online repositories. The names of the repository/repositories and accession number(s) can be found in the article/**Supplementary Material**.

## Supplementary material

Supplementary Figure 1 Development and validation of the AIDPI model. **(A)** Venn diagram of prognostic genes across the Puget 2012, Cavalli, Pomeroy, and CBTTC datasets. **(B)** Bar plot displaying feature coefficients for the Enet model (α = 0.1). **(C)** Determination of the optimal cut-off value for AIDPI using the surv_cutpoint function. **(D)** PCA plots showing gene expression data before and after batch correction across multiple datasets (Puget 2012, Cavalli, Paugh, Pomeroy, Witt, and CBTTC). **(E)** Kaplan–Meier survival curves demonstrating the prognostic significance of AIDPI across multiple datasets (Puget 2012, Cavalli, Paugh, Pomeroy, Witt, and CBTTC).

Supplementary Figure 2 Stratification of AIDPI values by clinical parameters. **(A)** Violin plots comparing AIDPI values across different tumor grades, genders, and stages. **(B)** Forest plot summarizing Cox regression analysis results for AIDPI values stratified by grade, age, and gender.

Supplementary Figure 3 Single-cell characterization of AIDPI-associated glioma cells. **(A)** tSNE plots of glioma, stromal, immune, and lymphocyte scores calculated using scGate and UCell. **(B)** Classification of immune and non-immune cells using CD45 expression with scGate. **(C)** CNV signal scatter plot derived from inferCNV, distinguishing normal and malignant glioma cells. **(D, E)** CNV heatmaps for reference T/NK cells and predicted normal glioma cells. **(F)** tSNE plot of cell clusters identified using resolution = 0.8. **(G–I)** Violin plots showing marker gene expression (SOX2 for glioma cells, CD3D for T cells, CD68 for myeloid cells). **(J)** tSNE plots illustrating patient-specific cell-type distributions.

Supplementary Figure 4 H1F0 copy number variations and expression across cancer cell lines. **(A)** Box plot showing H1F0 CNV alterations across cell lines in the CCLE database. **(B)** Box plot displaying H1F0 expression levels across CCLE cell lines.
