# Supplementary figures and images for: AI-derived prognostic model identifies high-risk gene signatures in pediatric gliomas
Source: Front Immunol. 2026 Mar 9;17:1704720. doi: 10.3389/fimmu.2026.1704720 (PMC13006634; doi:10.3389/fimmu.2026.1704720)

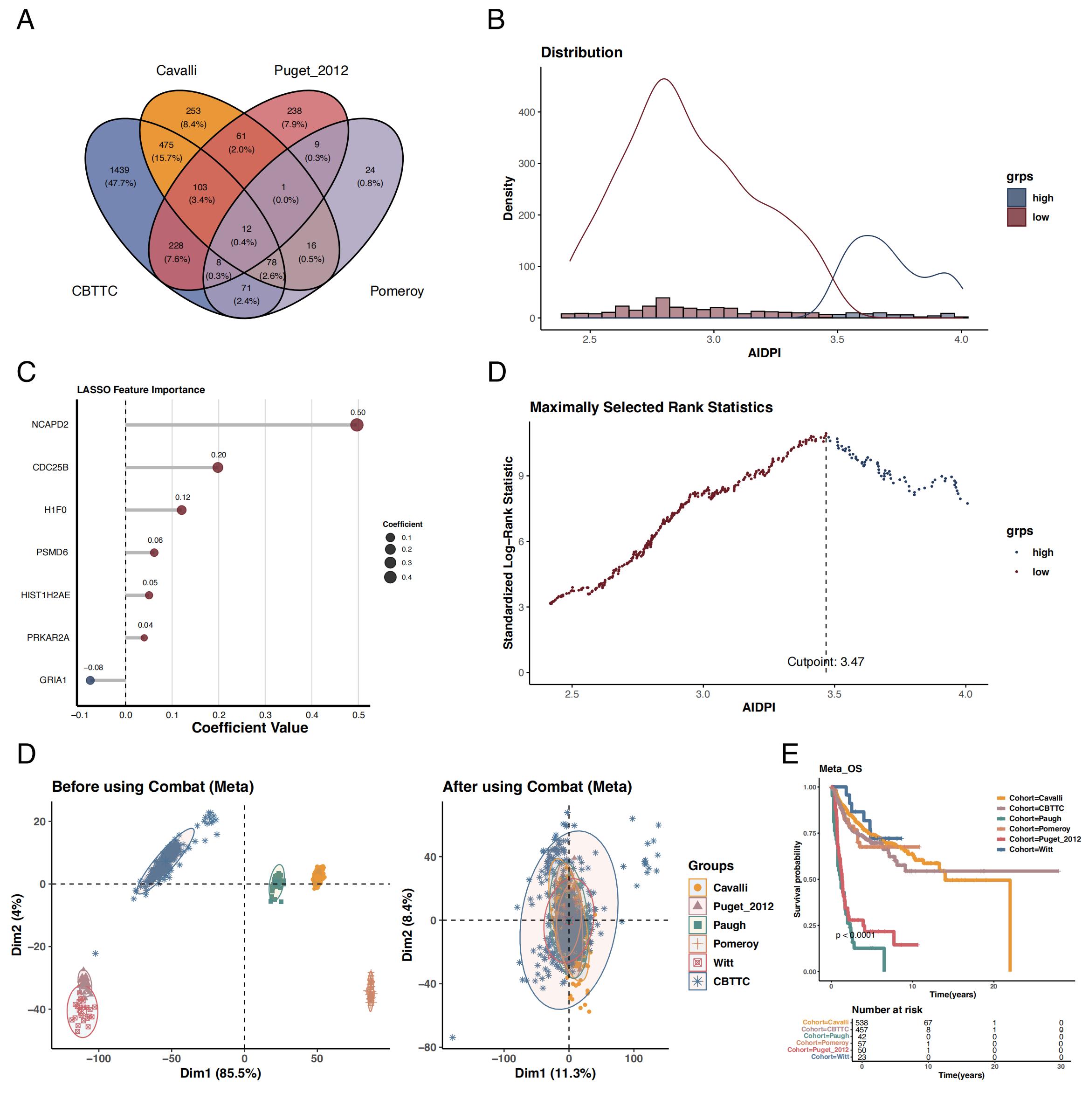

Supplement: Supplementary Figure 1 — Development and validation of the AIDPI model. (A) Venn diagram of prognostic genes across the Puget 2012, Cavalli, Pomeroy, and CBTTC datasets. (B) Bar plot displaying feature coefficients for the Enet model (α = 0.1). (C) Determination of the optimal cut-off value for AIDPI using the surv_cutpoint function. (D) PCA plots showing gene expression data before and after batch correction across multiple datasets (Puget 2012, Cavalli, Paugh, Pomeroy, Witt, and CBTTC). (E) Kaplan–Meier survival curves demonstrating the prognostic significance of AIDPI across multiple datasets (Puget 2012, Cavalli, Paugh, Pomeroy, Witt, and CBTTC). [file Image1.jpeg]

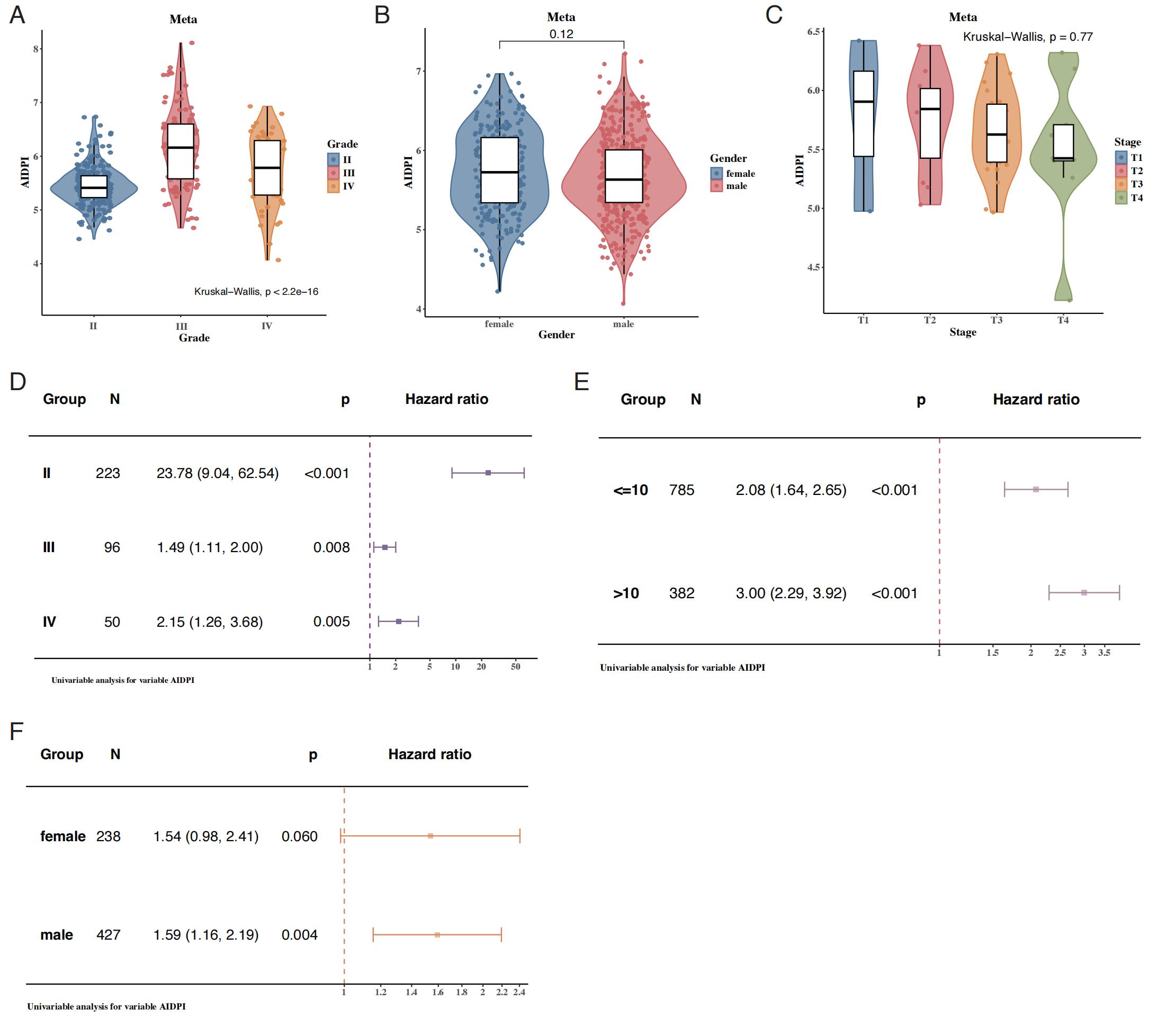

Supplement: Supplementary Figure 2 — Stratification of AIDPI values by clinical parameters. (A) Violin plots comparing AIDPI values across different tumor grades, genders, and stages. (B) Forest plot summarizing Cox regression analysis results for AIDPI values stratified by grade, age, and gender. [file Image2.jpeg]

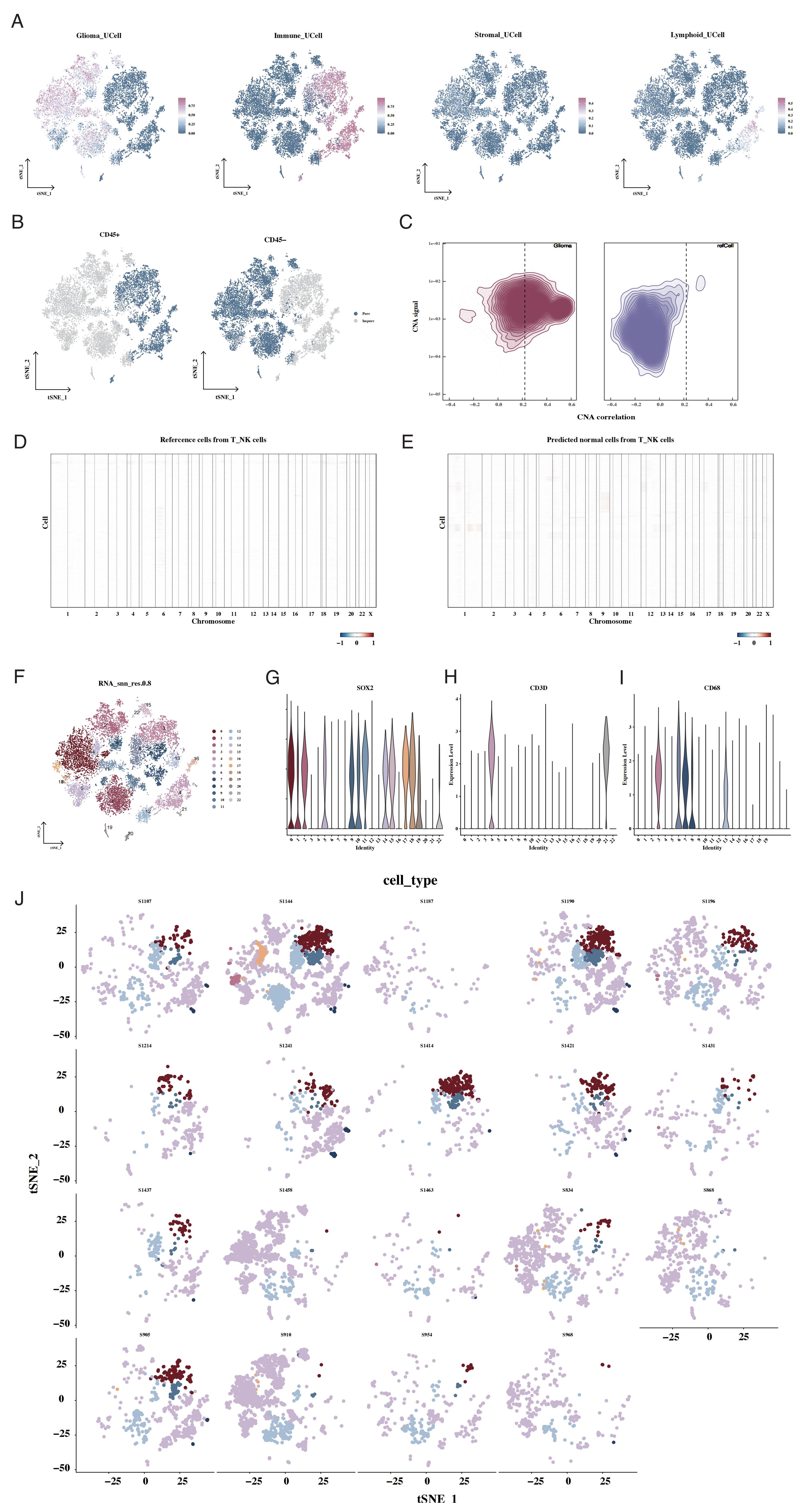

Supplement: Supplementary Figure 3 — Single-cell characterization of AIDPI-associated glioma cells. (A) tSNE plots of glioma, stromal, immune, and lymphocyte scores calculated using scGate and UCell. (B) Classification of immune and non-immune cells using CD45 expression with scGate. (C) CNV signal scatter plot derived from inferCNV, distinguishing normal and malignant glioma cells. (D, E) CNV heatmaps for reference T/NK cells and predicted normal glioma cells. (F) tSNE plot of cell clusters identified using resolution = 0.8. (G–I) Violin plots showing marker gene expression (SOX2 for glioma cells, CD3D for T cells, CD68 for myeloid cells). (J) tSNE plots illustrating patient-specific cell-type distributions. [file Image3.jpeg]

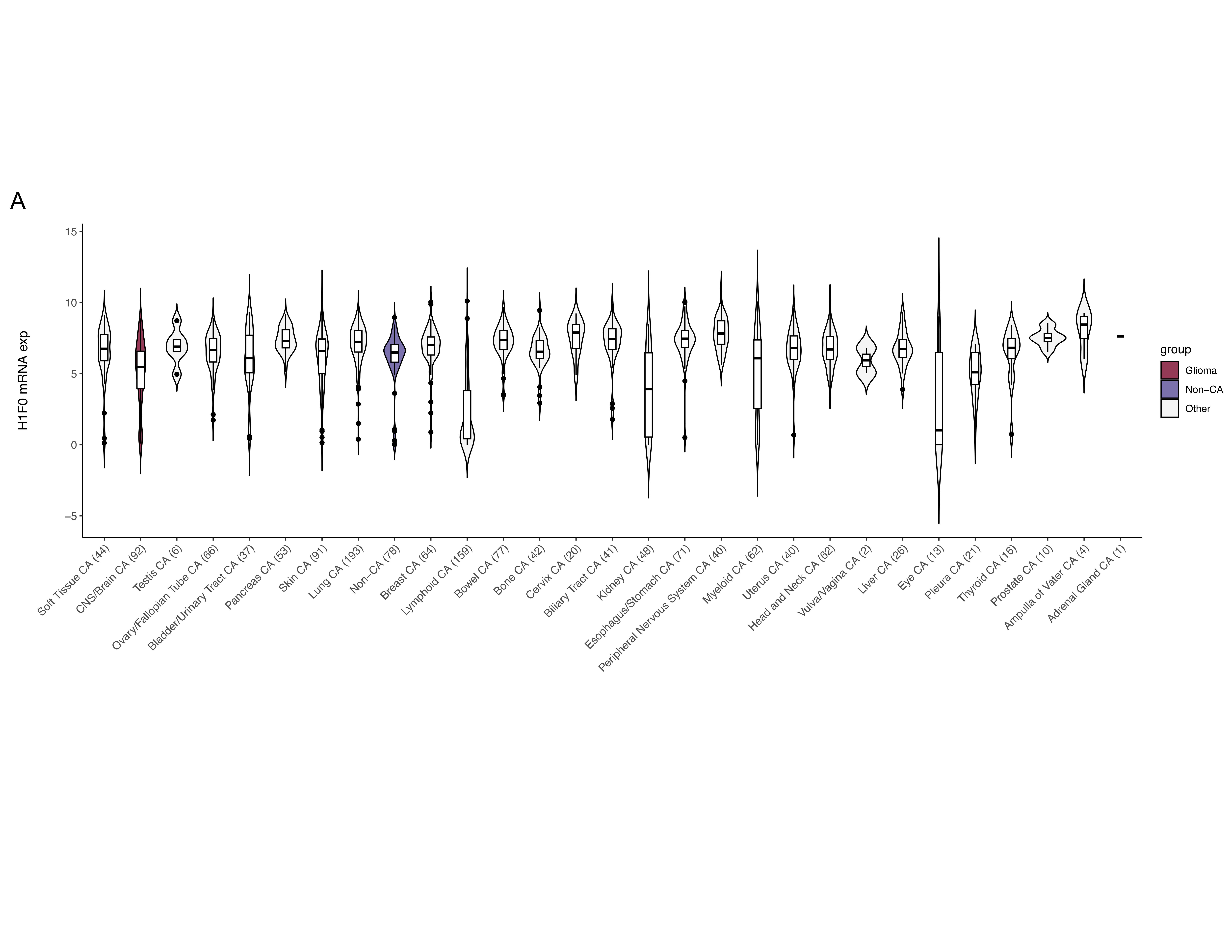

Supplement: Supplementary Figure 4 — H1F0 copy number variations and expression across cancer cell lines. (A) Box plot showing H1F0 CNV alterations across cell lines in the CCLE database. (B) Box plot displaying H1F0 expression levels across CCLE cell lines. [file Image4.jpeg]
