# Supplementary material for: AI-derived prognostic model identifies high-risk gene signatures in pediatric gliomas
Source: Front Immunol. 2026 Mar 9;17:1704720. doi: 10.3389/fimmu.2026.1704720 (PMC13006634; doi:10.3389/fimmu.2026.1704720)
Supplement: Supplementary file 5 [file Table1.docx]

**Supplementary table 1. A list of primers used in this study.**

| Gene | Forward sequence (5’ to 3’) | Reverse sequence (5’ to 3’) |
| --- | --- | --- |
| GAPDH | GGAGCGAGATCCCTCCAAAAT | GGCTGTTGTCATACTTCTCATGG |
| PRKAR2A | GTGCTGAGACCTATAACCCTGA | ACATGGCATCGAGAACTTGAGA |
